# Construction and Applications of Research Models for Intestinal Injury in Piglets: From In Vivo Models to In Vitro Models

**DOI:** 10.3390/ani16213458

**Published:** 2026-11-02

**Authors:** Pan Yang, Jiaman Pang, Bangli Yang, Yongqiang Liu, Min Song, Dun Deng, Yusheng Lu, Ting Rong

**Affiliations:** 1 Low Carbon Agriculture and Carbon Neutralization Research Center, State Key Laboratory of Swine and Poultry Breeding Industry, Institute of Animal Science, Guangdong Academy of Agricultural Sciences, Guangzhou 510640, China; ypan23@163.com (P.Y.); chestnuty1228@163.com (B.Y.); lyq990424@163.com (Y.L.); songmin@gdaas.cn (M.S.); dengdun@gdaas.cn (D.D.); 2 College of Animal Science and Technology, Southwest University, Chongqing 402460, China; pangjm@swu.edu.cn; 3 Institute of Agricultural Resources and Environment, Guangdong Academy of Agricultural Sciences, Guangzhou 510640, China; luyusheng1@gdaas.cn

**Keywords:** piglets, intestinal injury, intestinal epithelial cells, intestinal organoids, intestinal barrier

## Abstract

During weaning, piglets are exposed to multiple nutritional, environmental, pathogenic, and psychological stressors and are therefore susceptible to structural and functional intestinal injury. The resulting damage can lead to diarrhea, impaired growth, and increased susceptibility to disease, seriously compromising piglet health and the efficiency of pig production. Establishing stable and reliable experimental models that match specific research objectives is essential for elucidating the mechanisms of intestinal injury in piglets and for evaluating nutritional interventions and prevention and control strategies. Current research on porcine intestinal injury uses a multilevel framework centered on porcine intestinal epithelial cells, porcine intestinal organoids, and in vivo piglet models. These models offer distinct advantages in experimental controllability, tissue-level physiological relevance, and whole-organism biological fidelity, respectively, while each has a defined scope of application. Focusing on intestinal injury in piglets, this review comprehensively summarizes the construction methods, injury-induction approaches, and evaluation systems used across these model levels and analyzes the key factors affecting model stability and the comparability of research findings. This review is intended to inform mechanistic studies of intestinal injury in piglets, model standardization, and the evaluation of nutritional intervention strategies. On this basis, the review concludes that porcine intestinal epithelial cell lines are most appropriate for high-throughput mechanistic screening, organoids for questions that require epithelial diversity and donor- or region-specific context, and in vivo piglet models for confirming physiological and production relevance. Because model reproducibility depends strongly on challenge dose, animal or donor age, culture conditions, and assessment endpoints, a coordinated sequence from cell lines to organoids and finally to in vivo validation should be combined with standardized reporting of model parameters.

## Simple Summary

Piglets face abrupt changes in diet, housing, social contact, and exposure to disease at weaning. These stresses can damage the intestine, causing diarrhea, slow growth, and greater susceptibility to illness. Researchers use several experimental models to understand this damage and evaluate feeding or prevention strategies. This review compares three main approaches: pig intestinal cells grown in the laboratory, miniature three-dimensional intestinal tissues grown in the laboratory, and studies in piglets. Cell models are easy to control and useful for testing individual causes of injury, but they cannot reproduce the full complexity of the intestine. Three-dimensional tissue models more closely resemble the intestinal lining, although they do not include all immune, microbial, nerve, and blood-vessel interactions. Piglet models provide the greatest physiological and production relevance, but they require more time, expense, and animals, and their results may be influenced by several factors at once. The review explains how each model is established, how injury is produced and measured, and which questions each model can best answer. Using these models in a coordinated sequence may improve the reliability of intestinal health research and support better feeding and disease-prevention strategies in pig production. A practical sequence for combining these models, from cell-based screening to three-dimensional tissue models and finally to studies in piglets, is proposed to help researchers select the model that best matches each research question.

## 1. Introduction

Weaning is a critical stage of pig production during which intestinal homeostasis is profoundly disrupted. Piglets experience multiple stressors, including separation from the sow, transition from milk to solid feed, environmental change, and social regrouping. Because their digestive and mucosal immune systems are not yet fully mature, intestinal barrier function and immune homeostasis are particularly vulnerable. Weaning-associated stress can reduce feed intake, cause villus atrophy and crypt hyperplasia, and impair digestion and nutrient absorption. These changes are accompanied by disruption of tight junctions, increased intestinal permeability, intensified inflammatory responses, and microbial dysbiosis. The resulting outcomes include post-weaning diarrhea, impaired growth performance, and increased susceptibility to enteric pathogens [1,2,3]. In this review, intestinal injury is defined as a measurable disruption of intestinal structure or barrier function, including villus atrophy, tight-junction disruption, increased permeability, and epithelial cell death, whether it is caused by pathogen infection, chemical or mycotoxin exposure, oxidative stress, developmental abnormalities, or physiological stressors such as weaning. Models are included within this category when they reproduce at least one of these features; inflammation, intestinal dysfunction, or infection alone, without evidence of structural or barrier damage, is distinguished from intestinal injury as defined here. Intestinal injury is therefore both a major constraint on piglet health and production efficiency.

Stable and reproducible experimental models that match the research objective are essential for elucidating the mechanisms of intestinal injury in piglets and evaluating interventions. Current models use weaning stress, lipopolysaccharide (LPS), deoxynivalenol (DON), enterotoxigenic Escherichia coli (ETEC), dextran sulfate sodium (DSS), diquat (DQ), enteric viruses, and other challenges. These models reproduce different pathological processes, including inflammation, oxidative injury, toxin exposure, pathogen infection, and developmental abnormalities. However, their phenotypes are influenced by animal age, genetic background, husbandry conditions, challenge dose, administration route, and evaluation time. The lack of standardized modeling and assessment systems therefore continues to limit comparisons among studies.

Porcine intestinal epithelial cell models have become important tools for improving experimental control, resolving mechanisms, and screening functional interventions. Porcine epithelial cell lines such as IPEC-J2 and IPEC-1 form polarized epithelial structures and allow assessment of cell viability, transepithelial electrical resistance (TEER), paracellular permeability, tight-junction protein expression, inflammation, oxidative stress, and pathogen adhesion. Compared with animal experiments, cell models are less costly, provide more controllable conditions and higher reproducibility, and are amenable to high-throughput screening. They are particularly useful for examining specific signaling pathways and defining dose-time relationships [4,5]. Nevertheless, two-dimensional cell lines contain a single cell type, lack complex tissue architecture, and cannot reproduce the coordinated regulation of immune cells, extracellular matrix, and intestinal microorganisms. Their findings therefore require validation in higher-order models.

Advances in porcine intestinal organoid technology have provided a platform that bridges conventional cell models and animal experiments. Organoids derived from intestinal crypts or adult stem cells can self-renew and differentiate. In three-dimensional cultures or organoid-derived two-dimensional monolayers, they retain several epithelial lineages, including absorptive, goblet, enteroendocrine, and Paneth cells. Their cellular composition and functional characteristics therefore resemble the native intestinal epithelium more closely than those of a single cell line [6]. Porcine intestinal organoids have recently been used extensively to study infection by porcine epidemic diarrhea virus (PEDV), transmissible gastroenteritis virus (TGEV), rotavirus, and other enteric viruses. Basal-out, apical-out, and Transwell monolayer formats enable analyses of pathogen entry, cell tropism, barrier injury, and epithelial responses [7,8]. However, organoids remain predominantly epithelial and lack a complete immune, vascular, neural, and smooth-muscle microenvironment. Culture conditions, tissue source, and passage status also affect their maturity and stability. They cannot yet fully replace in vivo piglet models.

**Figure 1:**
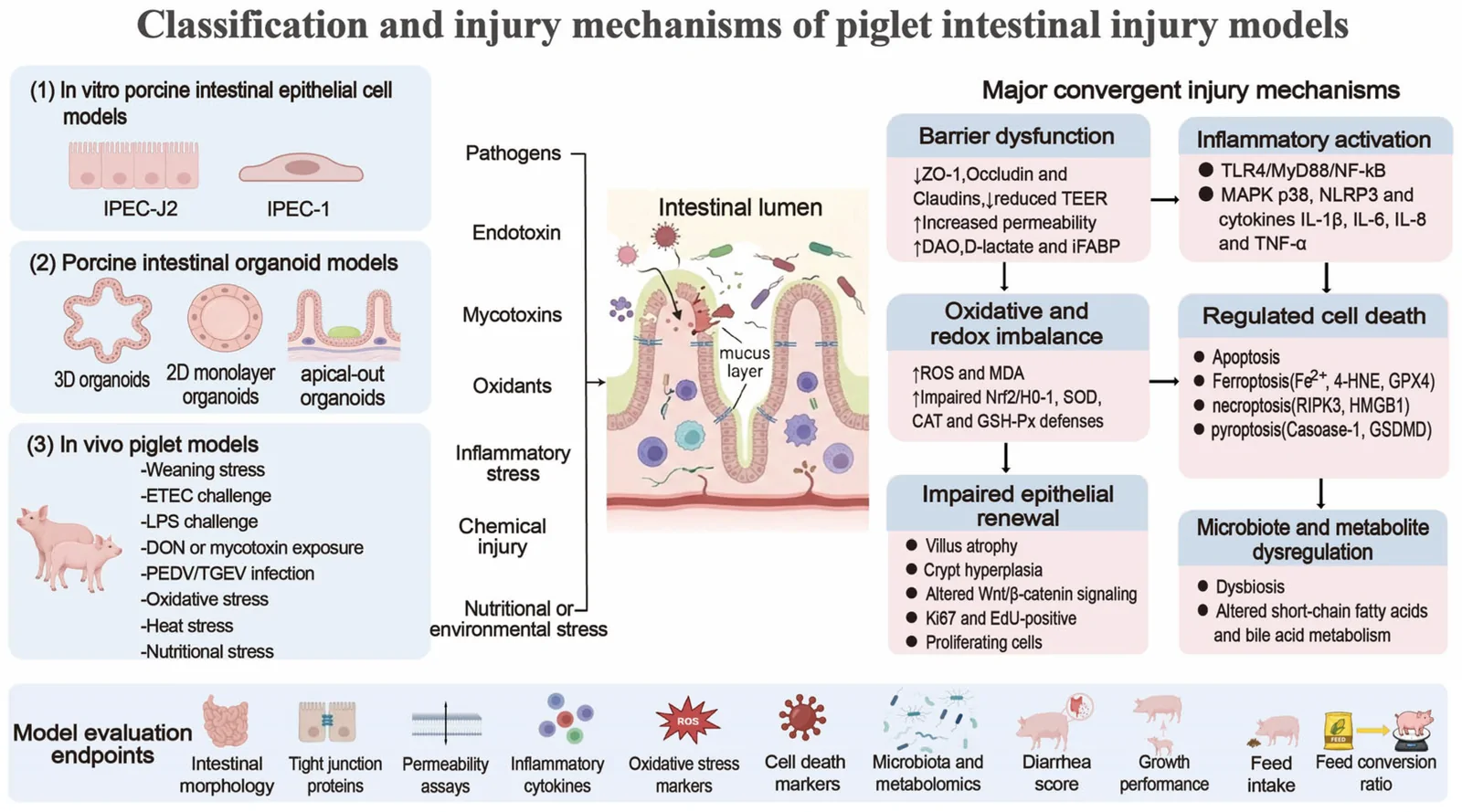
Classification and major injury mechanisms of piglet intestinal injury models.

Porcine intestinal epithelial cell lines, organoids, and in vivo piglet models represent complementary levels of experimental complexity and offer different balances of controllability, tissue complexity, and physiological fidelity (Figure 1). Cell models are suitable for mechanistic studies and initial screening of candidate interventions. Organoids can further resolve tissue-specific responses and cellular interactions, whereas in vivo models validate whole-animal physiological effects and production relevance. Most available studies focus on a single injury factor or model, and systematic comparisons of model construction, assessment criteria, and scope of application remain limited. Previous reviews have addressed weaning-associated intestinal health [1,2,3], porcine intestinal epithelial cell lines [4,5], or porcine intestinal organoids [6] separately, and an integrated comparison of all three model levels specifically for intestinal-injury research is still lacking. The present review aims to fill this gap. This review summarizes the principal experimental models used to study intestinal injury in piglets, including porcine intestinal epithelial cells, porcine intestinal organoids, and in vivo piglet models. It compares their construction methods, common injury-inducing factors, assessment systems, advantages, limitations, and appropriate applications to guide model selection in mechanistic and nutritional-intervention studies.

## 2. Literature Search Methodology

This article is a narrative review based on a structured literature search conducted on 21 October 2026 using Google Scholar, PubMed, Web of Science Core Collection, and Scopus. The main searches were restricted to English-language publications from January 2021 to October 2026. Two additional scoping searches without a year restriction were performed in Google Scholar to identify earlier methodological and review literature.

The search strategy addressed the three model levels covered by this review and combined the following concepts: for porcine intestinal epithelial cell models, (piglet* OR porcine OR swine) AND (IPEC-J2 OR IPEC-1 OR intestinal epithelial cell* OR enterocyte*) AND (injur* OR damage OR inflamm* OR barrier OR oxidative stress); for porcine intestinal organoid models, (porcine OR pig* OR swine) AND (intestinal organoid* OR enteroid*); and for in vivo piglet models, (piglet* OR weaned pig* OR weanling pig*) AND (intestinal injur* OR intestinal damage OR gut injur* OR intestinal barrier) AND (lipopolysaccharide OR LPS OR ETEC OR enterotoxigenic Escherichia coli OR deoxynivalenol OR mycotoxin* OR weaning OR rotavirus). These concepts were adapted to the syntax of each database. The Google Scholar keyword combinations piglet intestinal epithelial cell IPEC-J2 injury model, porcine intestinal organoids model, and weaned piglet intestinal injury model LPS ETEC yielded 33, 31, and 25 records, respectively. The corresponding Boolean searches returned 531, 131, and 340 hits in PubMed, 681, 167, and 924 hits in Web of Science (TS = topic searches), and 717, 167, and 444 hits in Scopus (TITLE-ABS-KEY searches). The two scoping searches were piglet intestinal injury model review and intestinal injury model establishment weaned piglets LPS review.

In total, 4191 records were retrieved across the four databases. After removal of duplicates by title, 1581 unique records remained. Records were screened by title and abstract, and eligible full texts were assessed against the inclusion criteria: English-language publications, published between 2021 and 2026, and describing the establishment, characterization, or application of porcine intestinal epithelial cell, porcine intestinal organoid, or in vivo piglet models of intestinal injury. Studies in non-porcine species, studies not addressing the intestine, and studies mentioning such models only peripherally were excluded. The searches in PubMed, Web of Science, and Scopus additionally served as cross-database verification of the core corpus; the additional records identified there either duplicated the core corpus or addressed peripheral topics and therefore did not change the final inclusion. The final corpus comprised 75 publications.

## 3. In Vivo Models of Intestinal Injury in Piglets

### 3.1. Weaning Stress Model

Weaning stress is a complex physiological process common in commercial pig production and an important in vivo model of intestinal dysfunction in piglets. Unlike in vitro models induced by a single stimulus, weaning combines separation from the sow, environmental change, social regrouping and hierarchy formation, and transition from liquid to solid feed (Table 1). These concurrent stressors intensify systemic stress responses and disrupt intestinal homeostasis. Circulating cortisol increases after weaning, activates inflammation-related signaling, and compromises barrier integrity. The resulting phenotype includes increased mucosal permeability, altered villus architecture, crypt hyperplasia, and impaired digestive and absorptive function [9]. Natural weaning can therefore serve as a physiological model of intestinal injury that reproduces weaning-associated dysfunction under production conditions.

Weaning-stress models generally use piglets weaned at 21–28 days of age. Comparisons between pre-weaning suckling controls and post-weaning samples collected at several time points allow dynamic assessment of changes in intestinal structure and function. Nutritional interventions can also be incorporated to determine how diet composition affects post-weaning intestinal adaptation. Transcriptomic comparison of jejunal and colonic tissues from 21-day-old suckling piglets and 35-day-old weaned piglets can reveal how this developmental transition reshapes intestinal epithelial gene expression [10]. However, such comparisons of 21-day-old suckling and 35-day-old weaned piglets differ in both weaning status and age. The intestinal epithelium undergoes substantial maturation during this period, including changes in digestive-enzyme activity, tight-junction organization, and mucosal immune development. Age-matched suckling controls at each post-weaning sampling point are therefore needed to separate the effects of weaning stress from normal developmental change [9,10]. Weaning-induced injury occurs mainly during the early post-weaning period, especially in the first week. Intestinal structure and function then gradually recover as feed intake adapts, the microbiota reorganizes, and the mucosa repairs [9]. Assessment therefore commonly integrates histology, barrier function, immune responses, and production performance. Villus height, crypt depth, and the villus-height-to-crypt-depth ratio in the jejunum and ileum characterize mucosal architecture. Expression of ZO-1, claudin-1, and occludin reflects barrier integrity, whereas goblet-cell abundance and mucus secretion indicate mucosal protection. Feed intake, average daily gain, diarrhea incidence, mucosal inflammatory mediators, and microbial composition provide complementary measures of the physiological impact of weaning stress [9,10].

The principal advantage of the weaning-stress model is its direct relevance to production. Weaning occurs naturally in every batch of piglets, requires no exogenous challenge, and realistically represents intestinal adaptation under commercial conditions. The model is therefore commonly used to evaluate the protective effects of amino acids, trace elements, probiotics, plant extracts, and other nutritional interventions during weaning. It also has limitations. Because several stressors occur simultaneously, the respective contributions of sow separation, dietary transition, and environmental change cannot be isolated precisely. Weaning injury is usually mild and partly self-resolving, so the model is unsuitable for severe pathological injury. In the absence of a pathogen challenge or another intensifying stimulus, individual differences in adaptation can obscure group effects and necessitate larger sample sizes [9].

### 3.2. LPS-Induced Model

The LPS-induced model is a commonly used model of inflammation and immune stress in piglets. It usually involves intraperitoneal injection of *Escherichia coli*-derived LPS to mimic the inflammatory response to endotoxin exposure during Gram-negative bacterial infection. LPS activates NF-κB and MAPK cascades through TLR4-MyD88-dependent signaling, promotes the release of proinflammatory cytokines, and induces mucosal inflammation, oxidative stress, and changes associated with cell death, including ferroptosis [11,12]. Unlike live-bacterial infection models, the LPS model avoids confounding by pathogen colonization, adhesion, and invasion. It is therefore well suited to studies of host inflammatory responses and anti-inflammatory interventions.

LPS models generally use either a single challenge or repeated challenges. A model may use a single high-intensity exposure, such as intraperitoneal injection of *Escherichia coli* O55:B5 LPS at 100 μg/kg body weight in 28-day-old weaned piglets 6 h before slaughter. This design captures rapid inflammatory and oxidative injury [11]. Models use repeated low doses to reproduce persistent inflammatory pressure during weaning. Intraperitoneal injections of 70 μg/kg body weight on experimental days 15, 18, and 21 can be used to assess the effects of prolonged immune stress on intestinal function and growth [12]. Functional compounds such as gamma-aminobutyric acid and plant polysaccharides are usually added to the diet before LPS challenge to evaluate their protective effects [11,12]. The LPS phenotype is strongly time dependent. Exposure increases serum inflammatory mediators within hours and is accompanied by oxidative stress and ferroptosis-related changes in the jejunal mucosa [11]. Repeated exposure gradually produces structural injury, growth impairment, and chronic inflammation over several weeks [12]. Single-challenge models are therefore appropriate for studying early inflammatory signaling, whereas repeated-challenge models address the longer-term effects of immune stress on intestinal homeostasis.

Assessment of LPS-induced injury covers inflammation, oxidative damage, cell death, and intestinal function. Common measures include TNF-α, IL-1β, and IL-6 in serum and jejunal mucosa; the activities of SOD, CAT, and GSH-Px and the concentration of MDA; ferroptosis-related indicators such as Fe^2+^, GPX4, SLC7A11, and ACSL4; jejunal villus height, crypt depth, and their ratio; and animal growth performance. The model provides a defined challenge, good reproducibility, and relatively simple procedures. It is useful for in vivo validation of anti-inflammatory and antioxidant nutritional interventions and for mechanistic studies of immune stress, particularly where live-pathogen experiments are impractical. However, intraperitoneal LPS bypasses the intestinal barrier and mainly models systemic endotoxemia rather than local intestinal infection. The intestinal lesions observed after intraperitoneal LPS are therefore largely secondary to systemic inflammation and circulating cytokines rather than to direct luminal exposure of the epithelium. This distinction from direct enteric infection should be kept in mind when judging model relevance and interpreting nutritional interventions: a compound that alleviates injury in this model may act mainly on systemic immune regulation rather than on local epithelial defense. It cannot reproduce pathogen adhesion, colonization, or invasion. The serotype, source, and batch of LPS may also alter biological activity.

### 3.3. Feed Toxin-Induced Injury Models

Mycotoxin contamination is a common feed-safety problem in pig production. Toxins such as DON enter pigs through contaminated feed and can cause sustained damage to the intestinal barrier. DON inhibits protein synthesis, induces oxidative stress, and disrupts tight junctions. These effects alter villus architecture, increase barrier permeability, and impair nutrient absorption [13]. Piglet mycotoxin-exposure models are therefore commonly used to investigate intestinal toxicity and to evaluate detoxifying agents, adsorbents, and nutritional interventions. Two main designs are used: high-dose exposure and prolonged low-dose feeding. Models generally administer a defined dose by gavage to induce rapid mucosal injury. Intestinal tissue collected 24 h after a single oral dose of DON at 0.2 mg/kg body weight in weaned piglets exhibits clear epithelial injury [13]. Long-term feeding of contaminated diets more closely resembles production conditions. Diets prepared with naturally mold-contaminated ingredients and measured toxin concentrations reproduce continued low-dose field exposure. Interventions can be added to the diet in advance. One example is the PPARγ agonist pioglitazone at 50 mg/kg feed for 14 days to assess protection against DON-induced barrier injury [13]. Exposure mode determines the timing and phenotype. DON exposure can damage villus tips in the duodenum and jejunum and reduce claudin-3/4 expression within 24 h [13]. In long-term contaminated-diet models, impaired digestion and absorption, altered barrier-gene expression, and reduced growth emerge gradually over several weeks. Evaluation includes villus height, villus necrosis and fusion, expression and localization of tight-junction proteins, permeability markers such as serum diamine oxidase and D-lactate, PPARγ transcriptional activity such as CD36 expression, and growth performance [13].

Feed toxin-induced models provide defined exposure conditions and relatively stable mechanisms, making them useful for studying mycotoxin-mediated barrier disruption and detoxification strategies. Nevertheless, different designs represent different toxicokinetic processes. A single high-dose gavage does not fully reproduce prolonged low-dose intake under production conditions, so extrapolation requires caution. Feeds are often co-contaminated with several mycotoxins, whereas single-toxin models cannot reproduce the full exposure profile. Subclinical doses also produce relatively mild injury and require sensitive molecular indicators for accurate assessment [13]. The doses used should also be interpreted against exposure under commercial feeding conditions. A single gavage of 0.2 mg/kg body weight represents acute exposure that exceeds the daily intake from feed contaminated near common regulatory guidance levels, whereas naturally contaminated diets deliver lower but sustained doses whose actual intake depends on feed consumption. Toxin purity and co-contamination also matter: purified DON isolates the effect of a single toxin, but moldy feed usually contains mixtures of mycotoxins whose combined effects may differ from those of DON alone. Exposure duration further determines whether adaptive responses or cumulative injury dominate. The phenotype of a toxin model should therefore always be interpreted together with the dose, toxin source and purity, co-contamination profile, feed intake, and exposure duration [13].

### 3.4. ETEC Infection Model

ETEC infection models usually use weaned piglets aged 21–28 days. Common strains used to establish reproducible diarrhea include O149:K91:K88ac, CVCC1502 (F4^+^), and F18 strains [14,15]. Other studies use a single high dose, such as 2.5 × 10^9^ CFU on day 19 or 1 × 10^9^ CFU/kg body weight [16,17]. Lower doses can reproduce milder infection [18,19]. To improve infection rates and consistency, some protocols include fasting for 8–12 h, streptomycin-mediated perturbation of the microbiota, neutralization of gastric acid with sodium bicarbonate, or selection by MUC4/F4 receptor genotype [14,20,21]. ETEC-induced injury follows a distinct temporal course. During the first 0–8 h, intestinal epithelial cells can undergo necroptosis and subsequently trigger inflammation in lamina propria immune cells [20]. Serum C-reactive protein, TNF-α, and IL-6 increase within 3–12 h [16]. Structural damage and systemic inflammation intensify as infection progresses and are usually evident 2–7 days after challenge. Compensatory defenses, including increased intestinal alkaline phosphatase (IAP) expression, may appear around day 5 [18]. Assessment integrates clinical signs, intestinal architecture, barrier function, and immunity. Diarrhea score and incidence are central indicators of model establishment. Histology focuses on villus changes in the jejunum and ileum, whereas ZO-1, occludin, and claudin-1 reflect barrier integrity. Serum diamine oxidase, D-lactate, and intestinal fatty acid-binding protein (i-FABP) indicate epithelial injury and permeability. Activation of TLR4-MyD88-NF-κB signaling, inflammatory mediators, bacterial translocation to mesenteric lymph nodes, and changes in the intestinal microbiota provide additional measures [14,16,17,18,19,20,21,22,23].

ETEC challenge produces a clear disease phenotype and closely reflects production problems. It is commonly used to study post-weaning diarrhea and evaluate probiotics, prebiotics, plant bioactives, antimicrobial peptides, and other alternatives to antibiotics. Large-scale experiments indicate that standardized challenge protocols can produce reproducible changes in diarrhea incidence and growth performance [22]. However, outcomes remain sensitive to strain, infection dose, piglet age, and host receptor background. The MUC4/F4 receptor genotype is a particularly important source of batch-to-batch variation in susceptibility [21]. Live-bacterial challenge also requires appropriate biosafety procedures and can raise animal-welfare concerns when infection is severe. Furthermore, F4 and F18 fimbriae depend on different host receptors, so results from the two types cannot be extrapolated directly [23,24].

### 3.5. Viral Infection Models

Enteric viral infections are major causes of diarrhea and intestinal dysfunction in piglets. PEDV, TGEV, and rotavirus display pronounced intestinal epithelial tropism. Unlike bacteria, which act mainly through adhesion, toxin release, and inflammatory activation, enteric viruses infect mature epithelial cells at the villus tips of the jejunum and ileum. Loss of absorptive enterocytes disrupts the brush border, ion transport, and nutrient absorption, causing osmotic and malabsorptive diarrhea [25]. Viral infection models are therefore used to investigate viral replication, epithelial injury, and host antiviral immunity.

Neonatal suckling piglets are commonly used because their intestinal epithelial cells are rapidly proliferating and maturing and are highly susceptible to enteric viruses. In one design, 3-day-old suckling piglets receive 15 mL of TGEV Miller or PEDV LJX at 1 × 10^7^ TCID_50_ mL orally, followed by tissue collection 3 days later to assess viral replication and intestinal injury [25]. Pharmacological simulation of host functional targets provides another strategy. Oral administration of the sodium/hydrogen exchanger 3 (NHE3) inhibitor tenapanor induces watery diarrhea, thinning of the intestinal wall, and villus shortening similar to viral infection. This approach tests the contribution of impaired ion transport to viral diarrhea [25]. In vivo piglet models of rotavirus and other enteric viruses are less widely used because their infection processes and host requirements are more complex. Recent mechanistic studies have increasingly used piglet-derived intestinal organoids [26]. Viral injury develops along a defined timeline. Viral replication and epithelial dysfunction begin early, followed within 24–72 h by rapidly increasing viral loads, diarrhea, villus atrophy, and impaired NHE3-mediated ion transport. Injury is often pronounced around day 3 [25]. Sampling schedules must therefore reflect the viral replication cycle and the course of tissue injury. Assessments include diarrhea scores, fecal water content, villus length and structural integrity in the jejunum and ileum, brush-border injury, viral nucleic acid abundance, infectious viral titers, and NHE3 mRNA expression, membrane abundance, and transport activity. Intestinal transcriptomics can further define host responses [25]. Viral infection models reproduce the development of viral diarrhea in piglets and are important for studying viral intestinal tropism, epithelial injury, and antiviral interventions. The high susceptibility of suckling piglets to PEDV and TGEV usually yields a clear and reproducible infection phenotype.

### 3.6. Other Injury Models

DSS is commonly used to induce chemical enteritis. It directly disrupts the epithelial mucus layer and tight junctions, compromises barrier integrity, and provokes inflammation. Unlike pathogen infection models, DSS chiefly reproduces mucosal injury and repair. It is therefore used to study epithelial regeneration, mucosal protection, and anti-inflammatory mechanisms [27]. In one protocol, 21-day-old piglets are acclimated for 7 days and then receive 5% DSS (molecular weight 36,000–50,000) by gavage for 4 days. The volume is 200 mL on day 8 and 100 mL daily on days 9–11 [27]. The model produces marked mucosal injury, including shortened jejunal villi, fewer goblet cells, and reduced epithelial proliferation. Evaluation includes intestinal histology, PAS staining of goblet cells, and expression of PCNA, β-catenin, and proteins involved in epithelial proliferation and repair [27]. The DSS model is simple to perform and allows adjustment of injury severity. It is suitable for studying epithelial repair and interventions that promote mucosal recovery. However, it is a noninfectious chemical injury model and differs from production-related disorders caused jointly by pathogens, nutritional change, and immune stress. Findings therefore require validation in other models.

DQ is a bipyridyl herbicide with strong redox activity, and its toxicity is driven mainly by excessive production of reactive oxygen species (ROS). In piglets, DQ causes oxidative stress, mitochondrial dysfunction, and inflammation. These effects disrupt epithelial barrier integrity, reduce tight-junction protein expression, increase intestinal permeability, and damage mucosal architecture. DQ-induced injury is commonly used to reproduce oxidative stress-related intestinal damage in weaned piglets and to evaluate antioxidant nutrients. Acute injury is generally induced by intraperitoneal injection. Xu et al. [28] used this model to examine whether glycine attenuates DQ-induced ferroptosis and barrier disruption. Similar models have evaluated plant polyphenols. Grape-seed proanthocyanidins have been tested after intraperitoneal injection of DQ at 10 mg/kg body weight by measuring intestinal structure, oxidative indicators, and barrier function [29]. DQ-induced injury is characterized by oxidative stress and barrier failure. Excess ROS promotes lipid peroxidation, reduces SOD and GSH-Px activities, and increases MDA. DQ also lowers ZO-1, occludin, and claudin-1 expression, increases epithelial permeability, and activates NF-κB and MAPK signaling, which aggravates mucosal injury. Recent work has linked this model to ferroptosis through changes in GPX4, SLC7A11, and other anti-ferroptotic molecules, providing a route to study the relationship between oxidative stress and cell death [28].

The low-birth-weight (LBW) piglet model is a developmental model based on a naturally occurring phenotype. It compares LBW and normal-birth-weight piglets without an exogenous challenge to examine the lasting effects of intrauterine growth restriction on intestinal development. One study compared piglets weighing approximately 724 ± 31 g at birth with normal-birth-weight piglets weighing approximately 1162 ± 98 g and collected intestinal tissue early after birth [30]. Intestinal developmental abnormalities in LBW piglets were closely associated with oxidative stress. Increased jejunal DUOX2 expression promoted endogenous ROS production, impaired villus development and brush-border enzyme activity, and disrupted intestinal angiogenesis. The model is commonly evaluated using ROS, MDA, and 8-OHdG; indices of mitochondrial function; and angiogenic markers such as CD31 and VEGF-A [30]. Its main advantage is direct relevance to commercial production because LBW piglets arise naturally in pig populations. It enables studies of how restricted fetal growth affects postnatal intestinal development, metabolic adaptation, and health. However, the LBW phenotype reflects genetic background, maternal nutrition, placental function, and the gestational environment.

**Table 1:** Comparison of intestinal injury models in piglets.

Model Type	Animals	Treatment	Injury Outcomes	Sampling Time	Reference
Weaning stress model	Weaned piglets, 21–28 days of age	Natural weaning without exogenous treatment; suckling piglets as controls	Villus height decreased, with crypt hyperplasia; serum cortisol increased; and gut permeability rose	-	[9]
Weaning stress	Suckling piglets at 21 d vs. weaned piglets at 35 d	-	Jejunal and colonic transcriptomes markedly remodeled after weaning	21 d (suckling) vs. 35 d (weaned)	[10]
LPS	Weaned piglets, 28 d	Single intraperitoneal injection of LPS (O55:B5) at 100 μg/kg body weight	Villus height and villus/crypt ratio decreased, goblet cells reduced, jejunal mucosal ferroptosis activated	6 h post-injection	[11]
LPS	Weaned piglets	LPS at 70 μg/kg body weight intraperitoneal injection on days 15, 18 and 21	Villus/crypt ratio decreased, IL-1β, IL-6 and TNF-α upregulated, growth retarded	15–21 d (repeated challenge)	[12]
DON	Weaned piglets	Single oral DON dose of 0.2 mg/kg body weight	Villus tip necrosis, claudin-3/4 downregulated, villus-crypt morphology impaired	24 h post-gavage	[13]
DON	Weaned piglets	Long-term feeding of diets formulated with moldy ingredients	Altered digestibility and barrier-gene expression, mild subclinical phenotype	after 14 d feeding	[13]
ETEC infection	Weaned pigs, 18–39 days of age	Oral F4/F18 strains at 10^6^–10^10^ CFU	Diarrhea rate increased peaking on days 2–7, villus atrophy, tight junctions disrupted	-	[24]
ETEC infection	Weaned piglets	Oral 15 mL of about 1 × 10^10^ CFU/mL daily for 3 days; fasting 8–12 h before challenge	Duodenal and ileal villus/crypt ratio decreased, ZO-1 and occludin downregulated in multiple segments	-	[14]
ETEC infection	Weaned piglets	20 mL of 5 × 10^9^ CFU/mL daily for 4 days	Diarrhea with intestinal immune dysregulation, disturbed bile acid metabolism, NF-κB-driven inflammation activated	-	[15]
ETEC infection	Weaned piglets	Single oral dose of 2.5 × 10^9^ CFU on day 19	Plasma GSH-Px and CAT activities decreased, MDA increased, jejunal epithelial apoptosis rate increased	-	[17]
ETEC infection	Weaned piglets	Oral dose of 1 × 10^9^ CFU/kg body weight	Serum CRP, TNF-α and IL-6 increased, crypt depth increased, villus/crypt ratio decreased	-	[16]
ETEC infection	Weaned piglets	Single gavage of 6 mL at 10^6^ CFU/mL	Diarrhea incidence increased with mild phenotype	-	[19]
ETEC infection	Nursery pigs	8 × 10^8^ CFU	Intestinal alkaline phosphatase markedly upregulated on the small-intestinal villus surface	d 5 post-challenge	[18]
ETEC infection	Weaned piglets	Oral ETEC CVCC196 at 1 × 10^10^ CFU/kg body weight after streptomycin pretreatment	Plasma i-FABP increased, crypt epithelial necroptosis followed by lamina propria cell pyroptosis	0–8 h post-challenge	[20]
ETEC infection	Weaned piglets (MUC4-susceptible genotype)	Pre-screening of MUC4/F4 receptor-susceptible individuals before challenge	Tight-junction proteins downregulated, GSH, SOD1 and HO-1 decreased, MDA increased	-	[21]
ETEC infection	Weaned piglets	Oral 100 mL of 1 × 10^8^ CFU/mL ETEC K88 for 3 days	The diarrhea rate was 85.71% versus 44.64% in controls; serum T-SOD decreased, and MDA increased	-	[22]
ETEC infection	Weaned piglets	Oral 10 mL of ETEC F18 at 1 × 10^10^ CFU/mL daily for 3 days; fecal consistency score (0–3 scale) as the modeling success criterion	Diarrhea scores increased, villus height decreased, crypts deepened	-	[23]
TGEV/PEDV infection	3-day-old suckling piglets	Oral 15 mL of 1 × 10^7^ TCID_50_/mL TGEV or PEDV, necropsy 3 days later	Watery diarrhea developed with villus atrophy, NHE3 activity and membrane abundance decreased	3 d post-inoculation	[25]
Pharmacological diarrhea model (tenapanor, NHE3 inhibitor)	Suckling piglets	Oral NHE3 inhibitor Tenapanor to reproduce watery diarrhea	Reproduced the viral phenotype with watery diarrhea, intestinal bulging, wall thinning and villus shortening	-	[25]
DSS	Weaned piglets	After 7-d pre-feeding, 5% DSS gavage for 4 days	Jejunal villi shortened, goblet cells reduced, proliferation proteins such as CD24 and cyclin D1 downregulated	after 4-d gavage (d 11)	[27]
Low birth weight	Naturally occurring LBW piglets (724 ± 31 g vs. 1162 ± 98 g)	-	Jejunal villus height and maltase activity lower, ROS and MDA higher, intestinal angiogenesis impaired	early after birth	[30]
Weaning plus ETEC	Weaned piglets	After a 14-d feeding trial, oral 10 mL of about 1 × 10^9^ CFU/mL ETEC K88	Diarrhea rate increased, jejunal villus height decreased, GSH, T-SOD and GSH-Px decreased	-	[31]

## 4. Porcine Intestinal Epithelial Cell Injury Models

### 4.1. Origins and Biological Characteristics of Porcine Intestinal Epithelial Cell Models

IPEC-J2 is a commonly used porcine intestinal epithelial cell model. Berschneider established the line in 1989 from the jejunum of a neonatal piglet [4,32]. IPEC-J2 is nontransformed and was not immortalized with a viral oncogene. It therefore retains some structural and functional features of primary porcine intestinal epithelial cells [4,32]. IPEC-J2 cells form apical microvilli and express tight-junction proteins including claudin-1/2/3/4/5/7/8, occludin, and ZO-1. They constitutively express pattern-recognition receptors TLR1-6 and TLR8-10, secrete inflammatory mediators such as IL-6, IL-8, and IL-18, and express the mucins Muc1 and Muc3 [4,32]. These properties allow IPEC-J2 cells to reproduce aspects of barrier regulation and immune responses to LPS, pathogens, and toxins, supporting their widespread use in porcine intestinal health, nutritional regulation, and feed-additive assessment. The line nevertheless has biological limitations. It consists mainly of one jejunal epithelial-like cell type and lacks the complete multicellular composition of the intestine, particularly goblet cells, which limits mucus secretion. MUC2 and the enteroendocrine marker CHGA are undetectable at both transcript and protein levels, and lineage markers such as lysozyme and alkaline phosphatase are expressed at much lower levels than in primary intestinal epithelial cells [32,33]. IPEC-J2 can therefore model selected barrier and inflammatory responses but cannot replace the complex in vivo intestinal microenvironment. Other porcine intestinal epithelial cell models include IPEC-1 and IPI-2I. IPEC-1 is another nontransformed line derived from the jejunal-ileal region of a neonatal piglet [32]. Although it is less widely used than IPEC-J2, it has served as a neonatal porcine intestinal model in studies such as bacteriophage intervention against ETEC [34]. By contrast, IPI-2I was immortalized by transfection with the pSV3-neo plasmid, which carries simian virus 40 large T antigen and a neomycin-resistance gene. Immortalization may alter normal physiological functions [32].

The barrier phenotype of IPEC-J2 is not fixed and is strongly influenced by the culture system. Under conventional conditions containing 5–10% fetal bovine serum, TEER can exceed 1000 Ω·cm^2^ and may reach more than 2000 Ω·cm^2^. With 10% porcine serum, TEER decreases to 200–400 Ω·cm^2^. Culture in Wnt3a/R-spondin/Noggin-conditioned medium can reduce TEER further to approximately 50 Ω·cm^2^, closer to the resistance of small-intestinal epithelium in vivo [4]. These differences arise because TEER is sensitive to passage number, seeding density, medium formulation, serum source and concentration, and membrane conditions. TEER rises as monolayers mature after seeding and varies with passage number, so a defined passage window and post-seeding culture period are required before measurement. High seeding density accelerates monolayer formation but also overgrowth and subsequent barrier decline. Serum composition is a major factor: fetal bovine serum supports high-resistance monolayers, whereas porcine serum yields lower TEER that may better reflect in vivo conditions. Medium supplements, such as Wnt-pathway modulators, and the pore size and coating of Transwell membranes further shift absolute values. TEER should therefore be compared only within identical culture conditions, and passage number, cell density, medium, serum, and membrane specifications should be reported [4,35]. A study of berberine by Cornelius et al. [35] also showed that cell models from different species or lineages can produce divergent or even opposite results. Berberine has been reported to enhance barrier function in human Caco-2 and related models, but in IPEC-J2 it caused dose-dependent barrier injury and apoptosis [35]. Cell models used to evaluate nutritional additives, functional compounds, or intestinal protection in pigs should therefore be matched to the target species.

### 4.2. LPS-Induced Inflammation and Barrier Injury Models

LPS, a major component of the outer membrane of Gram-negative bacteria, activates NF-κB and MAPK inflammatory cascades through Toll-like receptor 4 (TLR4) and MyD88-dependent signaling. It promotes proinflammatory cytokine release and disrupts tight junctions and epithelial barrier function. LPS challenge is therefore a commonly used noninfectious approach for inducing intestinal epithelial inflammation in vitro. Reported concentrations in IPEC-J2 range from 1 μg/L to 40 μg/mL, and exposure times range from 2 to 48 h. Parameters must be selected for the intended phenotype. Brief, low-intensity exposure mainly activates inflammatory signaling, whereas structural barrier injury generally requires longer exposure. Zhao et al. [36] treated IPEC-J2 cells with 10 μg/mL LPS for 2–6 h. TNF-α and IL-8 secretion and phosphorylation of proteins in the TLR4/IRAK1/TRAF6/NF-κB p65 axis increased significantly, but TEER did not decline within 6 h. Capsaicin pretreatment suppressed the inflammatory cascade and improved tight-junction protein localization. This result suggests that brief LPS exposure preferentially elicits inflammation and that barrier dysfunction requires longer stimulation. Xie et al. [37] treated IPEC-J2 cells with 10 μg/mL LPS for 48 h and observed increased apoptosis, caspase-3/8/9, IL-1β, and TNF-α. Porcine β-defensin 129 partly attenuated these effects. Li et al. [38] used the same dose for 16 h and conducted transcriptomic analysis to confirm the central roles of MAPK and NF-κB signaling. Sea-buckthorn polysaccharides reversed these inflammatory responses in a dose-dependent manner. LPS can also elicit other forms of cellular stress. A brief 6-h low-dose exposure can induce ferroptosis by reducing GPX4 and SLC7A11 expression, whereas indole-3-carbinol suppresses this process through the AhR-Nrf2-SLC7A11-GPX4 axis [39]. Wang et al. [11] selected a model that reduced viability to approximately 80% and identified 40 μg/mL LPS for 48 h as an IPEC-J2 injury condition. The LPS-induced IPEC-J2 model is inexpensive, simple, and reproducible and is commonly used to study porcine intestinal inflammation and functional interventions. However, studies vary substantially in LPS source, serotype, reagent specification, dose, and exposure time.

### 4.3. Mycotoxin-Induced Injury Models

DON is among the most frequently detected mycotoxins in feed, and pigs are particularly sensitive to it. DON-induced IPEC-J2 models are commonly used to evaluate effects on the intestinal epithelial barrier and potential protective interventions. Li et al. [13] showed that DON reduced tight-junction protein expression, increased paracellular permeability, and inhibited PPARγ transcriptional activity in a dose-dependent manner. A PPARγ agonist maintained barrier integrity by suppressing endocytic degradation of claudin-4, whereas this protection disappeared in PPARγ-knockout cells. These results implicate PPARγ signaling in DON-mediated barrier injury. Huang et al. [40] selected a sublethal model based on a decline in viability below 70% and used 0.5 μg/mL DON for 24 h. Selenomethionine attenuated DON-induced oxidative injury by activating an Nrf2-related antioxidant pathway independently of MAPK signaling. Zearalenone (ZEA) at 20 μg/mL for 24 h increased the number of ROS-positive cells and caused injury through an oxidative stress-mitochondrial apoptosis-NF-κB/NLRP3 inflammatory axis [41]. Fumonisin B_1_ at 20 μmol/L for 48 h reduced viability and TEER, induced apoptosis, and downregulated tight-junction proteins in a dose-dependent manner. Resveratrol protected cells through Nrf2 activation, and combined exposure to FB_1_, FB_2_, and FB_3_ produced synergistic toxicity [42]. Huang et al. [40] selected DON from viability responses. Other studies used field contamination levels [43], whereas the FB_1_ study used regulatory limits and published concentrations [42]. Some studies also include acute in vivo exposure to strengthen the connection between cellular models and animal exposure [13].

Mycotoxin-induced IPEC-J2 models show clear dose–response relationships and good reproducibility. They are valuable for investigating intestinal toxicity and screening detoxifying agents and antioxidant nutritional interventions. Most studies, however, assess barrier injury mainly through tight-junction protein expression and less often measure TEER or permeability.

### 4.4. Viral and Other Pathogen Infection Models

#### 4.4.1. ETEC Infection Model

ETEC is a major enteric pathogen associated with post-weaning diarrhea in piglets. Its fimbrial adhesins, including F4/K88 and F18, bind receptors on small-intestinal epithelial cells. ETEC then secretes heat-labile enterotoxin, heat-stable enterotoxins (STa and STb), EAST-1, and other virulence factors. These products disrupt ion transport and fluid balance and promote epithelial barrier injury, inflammation, and microbial dysbiosis [24].

IPEC-J2 is a commonly used in vitro model of ETEC pathogenesis and intervention. Typical conditions use approximately 10^6^ CFU/mL or an MOI of 10–100 for 1–3 h. Li et al. [44] tested Bacillus licheniformis PF9 at probiotic-to-pathogen ratios from 1:1 to 1:10 and found dose-dependent protection against ETEC-induced loss of TEER and activation of TLR4-NF-κB signaling. Fu et al. [45] combined ETEC challenge with an NF-κB inhibitor to establish that β-defensin 118 exerts anti-inflammatory and anti-apoptotic effects. Shen et al. [46] removed bacteria after a 1-h infection and measured responses at several later time points. This design revealed dynamic regulation of the TLRs-TAK1-NF-κB/MAPK axis by pBD2. Host genetic background also affects model outcomes. Chen et al. [47] reported that their IPEC-J2 cells originated from a pig with an MUC4 genotype associated with F4 resistance. Donor variation may therefore alter ETEC adhesion and downstream outcomes, and studies should report cell source and receptor genotype. A time-course model developed by Hu et al. [48] showed that ETEC-K88 adhesion increased after 4 h and peaked at 24 h, with 4–8 h representing a potentially critical interval for host responses and intervention. Other porcine epithelial lines have also been used. IPEC-1 cells supported evaluation of encapsulated bacteriophages against ETEC O9:H9 [34]. ETEC infection models reproduce key interactions between the pathogen and porcine intestinal epithelium, but their parameters vary with bacterial virulence, receptor expression, and infection conditions.

#### 4.4.2. Other Bacterial and Toxin Models

Clostridium perfringens type C and its major virulence factor, Clostridium perfringens beta2 toxin (CPB2), are important causes of necrotizing enteritis in neonatal piglets. CPB2 directly injures intestinal epithelial cells and induces inflammation and apoptosis. IPEC-J2 models challenged with purified CPB2 are therefore used to examine its enterotoxic mechanisms. Compared with whole-bacterium infection, a purified-toxin model reduces confounding from bacterial adhesion, proliferation, and other virulence factors and better isolates the host response to a single toxin. Li et al. [49] treated IPEC-J2 cells with purified CPB2 at 20 μg/mL for 24 h and combined S100A9 knockdown with transcriptomic analysis. They identified S100A9 as a key regulator of CPB2-induced inflammation and a potential molecular node in the inflammatory effects of C. perfringens toxins. CPB2-induced injury also involves apoptosis. Yang et al. [50] classified animals as sensitive or resistant using diarrhea scores and found that FTO-mediated m^6^A demethylation regulates CPB2-induced apoptosis in IPEC-J2 cells by altering caspase-3 mRNA stability. This mechanism may contribute to individual differences in toxin sensitivity. The CPB2-induced IPEC-J2 model is useful for examining the direct epithelial effects of a major C. perfringens toxin and for screening anti-inflammatory and anti-apoptotic targets. It nevertheless represents a single toxin and cannot reproduce bacterial colonization, microbial changes, or cooperation among multiple virulence factors during natural infection. Interpretation of necrotizing enteritis therefore requires complementary animal infection models and assessment systems.

### 4.5. Oxidative and Nutritional Stress Models

Weaning stress is not caused by one stimulus but reflects interacting neuroendocrine stress, dietary antigens, redox imbalance, and microbial signals. These factors converge on pattern-recognition receptor activation, inflammatory cascades, Nrf2-mediated redox regulation, and tight-junction disruption to alter epithelial homeostasis [9]. Models based on individual oxidative or nutritional stressors are commonly used to dissect weaning-associated barrier injury and evaluate potential protection.

Hydrogen peroxide (H_2_O_2_) is a commonly used in vitro inducer of oxidative stress and reproduces intestinal epithelial injury caused by excessive ROS. Studies generally define sublethal conditions from reductions in cell viability, but doses vary. Lu et al. [51] selected 1 mmol/L H_2_O_2_ for 1 h, which reduced viability to approximately 53%, and used this model to evaluate soybean-peptide protection through Nrf2 signaling. Li et al. [52] used 0.4 mmol/L H_2_O_2_ for 1 h, which reduced viability to approximately 52%, to examine apoptosis through the mitochondrial and death-receptor pathways. The difference in dose indicates that medium composition, serum conditions, and passage status can affect sensitivity. Other oxidants are also used. Tert-butyl hydroperoxide at 200 μmol/L for 3 h induces oxidative stress in IPEC-J2 and supports evaluation of protective mechanisms [53]. The free-radical generator 2,2′-azobis(2-amidinopropane) dihydrochloride produces sustained ROS accumulation and is used to test antioxidant regulation of oxidative stress and barrier function [54]. Infection and oxidative stress also interact. Jin et al. [21] showed that ETEC F4 suppresses epithelial Nrf2 nuclear activation, reduces glutathione synthesis, and thereby intensifies oxidative stress and barrier injury.

Nutritionally mediated oxidative injury has also received increasing attention. Ferroptosis, a form of cell death associated with lipid peroxidation, offers a framework for studying nutritional oxidative stress. Liu and Lin [55] treated IPEC-J2 cells with a fatty-acid mixture at a total concentration of 200 μmol/L and varied the n6: n3 ratio from 1:1 to 20:1. Increasing the proportion of n-6 fatty acids intensified lipid peroxidation and ferroptosis. Abnormal endogenous ROS generation can also contribute to epithelial injury. Zou et al. [30] found that increased jejunal DUOX2 expression in LBW piglets promoted ROS production and altered intestinal angiogenesis through MMP3. Nutritional stress models also reproduce epithelial responses to plant-protein antigens and dietary changes after weaning. Wang et al. [56] treated IPEC-J2 cells with 5 mg/mL soybean 7S globulin (β-conglycinin) for 24 h to model sensitivity to dietary antigens. DSS can model epithelial injury and repair in vitro. Treatment with 0.08% DSS for 24 h has been used to test how lactate promotes epithelial proliferation and repair through Wnt/β-catenin signaling [27].

Environmental challenges can model additional components of the complex stress experienced during weaning. Perruchot et al. [57] developed a two-stage heat-stress and recovery model in which IPEC-J2 cells were cultured at 41 °C for 5 days and then at 37 °C for 5 days. Heat stress suppressed barrier function and mucus secretion. Most measures recovered after return to 37 °C, but MUC2 secretion and antioxidant capacity remained impaired, indicating that short-term recovery did not fully reverse functional injury. Xu et al. [58] modeled the hyperosmotic porcine intestinal lumen by adding 150 mmol/L NaCl to raise culture osmolality to 600 mOsm. Betaine reduced mitochondrial apoptosis and autophagy-related injury as an osmoprotectant. Oxidative and nutritional stress models reproduce weaning-associated epithelial injury through ROS accumulation, cell death, and barrier disruption. Compared with inflammation-only models, they better represent noninfectious contributors to disrupted intestinal homeostasis during weaning. However, studies vary in dose, duration, and assessment criteria, and no single stressor can reproduce the complex in vivo environment.

### 4.6. Advantages, Limitations, and Appropriate Uses of Cell Models

Representative cell-based injury models, including their experimental conditions and main outcomes, are summarized in Table 2. Porcine intestinal epithelial cell models offer physiological relevance, mechanistic tractability, and experimental convenience. IPEC-J2 and IPEC-1 retain selected biological features of porcine intestinal epithelium and respond to porcine pathogens and feed-associated compounds more similarly to the target animal than do heterologous lines. In vitro systems also support pathway inhibitors, small interfering RNA, gene knockout, reporter assays, and other molecular tools for causal testing of specific signaling pathways. Their low cost, short experimental cycle, and high throughput make them useful for initial screening of functional additives and detoxifying agents. Important limitations remain. Conventional cell lines have a simple cellular composition and lack a mature mucus layer and the multicellular architecture of the intestine. Insufficient expression of MUC2, CHGA, and related markers restricts their capacity to model complex intestinal functions. Barrier measures such as TEER are sensitive to serum source, medium composition, and culture conditions, which limits comparison of absolute values among laboratories. Some intestinal viruses, including clinical PEDV isolates, infect IPEC-J2 inefficiently. Because IPEC-J2 originates from the jejunum, it also cannot fully represent functional differences among intestinal segments.

## 5. Porcine Intestinal Organoid Injury Models

### 5.1. Tissue Sources, Culture, and Characterization of Organoids

Porcine intestinal organoids are derived mainly from adult stem cells in intestinal crypts and depend on their capacity for self-renewal and differentiation within a defined niche. Stable and broadly applicable porcine embryonic stem cell (ESC) and induced pluripotent stem cell (iPSC) systems have not yet been established (Table 3). Pluripotent-stem-cell differentiation into organoids therefore remains immature in pigs, and current models rely mainly on adult crypt cultures [6,60]. Organoids have been established from pigs of different ages and genetic backgrounds [26,33,61,62,63,64]. Sources include the duodenum, jejunum, ileum, colon, and multiple ileal regions [65]. This diversity preserves developmental and segment-specific features and enables studies of age, intestinal region, and host genetics. Endoscopic biopsy further increases flexibility by allowing repeated sampling from the same donor and derivation of organoids from diseased tissues [60].

Establishment of porcine intestinal organoids generally involves crypt isolation, embedding in a three-dimensional matrix, and culture with defined growth factors. Crypts are usually isolated by mild EDTA chelation, with DTT used to remove mucus. Conditions are optimized for intestinal region and include shaking at 4 °C with 10 mmol/L EDTA and 1 mmol/L DTT or treatment at room temperature with 9 mmol/L EDTA and 3 mmol/L DTT [6,66]. The culture system reproduces niche signals required for stem-cell maintenance. Wnt3a, R-spondin1, and Noggin are central to proliferation and organoid formation. EGF, N2/B27, nicotinamide, N-acetylcysteine, and modulators of p38, TGF-β, GSK-3, and ROCK signaling are commonly added to support expansion and stability [6,33,60,63]. Common platforms include commercial media such as IntestiCult, L-WRN-conditioned medium, and laboratory-defined formulations [26,33,60,63]. In-house conditioned media can be functionally assessed with TOPFlash and ELISA, reducing cost while improving batch control [60]. Culture requirements vary among intestinal regions. Colonic organoids often require prostaglandin E2 and gastrin for stable growth [67]. Organoids are commonly passaged every 4–7 days at a 1:3 to 1:4 split ratio using TrypLE digestion and mechanical dissociation. High establishment rates after recovery of cryopreserved crypts make standardized organoid biobanks feasible [63,66].

Reliable organoid models require characterization of cell-lineage composition, spatial architecture, barrier function, and molecular fidelity to the source tissue. Lineages are usually assessed by qPCR and immunofluorescence. LGR5, HOPX, and BMI1 identify stem-cell populations; Ki67 and SOX9 indicate proliferation; LYZ, MUC2, and CHGA mark Paneth, goblet, and enteroendocrine cells; and VIL1 and ALPI identify absorptive epithelial cells [26,33,60,63]. Apical ZO-1 localization, brush-border villin, and cystic or budding morphology establish epithelial polarity and tissue architecture [26,68]. Retention of tracers such as FITC-dextran in the lumen, or leakage across a monolayer, can measure barrier function [63]. Molecular fidelity is also important. Adult porcine jejunal organoids show high transcriptomic concordance with their source tissue, with correlations reaching 0.98 [63]. Approximately 48% of genes differentially expressed between jejunum and colon remain stably different in organoids, indicating partial retention of regional identity [10]. Organoids can also preserve donor-specific regulatory variation, including differences in the glycosylation genes FUT2 and B4GALNT2, enabling investigation of genetic effects on intestinal responsiveness [64].

Despite their tissue relevance, porcine intestinal organoids remain difficult to characterize and standardize. A major constraint is the limited availability of pig-specific antibodies. Reliable cross-reactive antibodies are lacking for markers such as LGR5 and CAII, so some studies depend on indirect molecular characterization by qPCR [60]. Tissue source, donor age, and culture system can also alter organoid maturity and function. More standardized culture and characterization systems are therefore needed.

### 5.2. Approaches for Establishing Organoid Injury Models

The culture configuration determines how an organoid injury model can be challenged because epithelial polarity controls access to the target surface. Conventional three-dimensional organoids are basal-out, with the apical surface enclosed within the lumen. Exogenous agents therefore cannot readily contact the apical epithelium. Apical exposure can be achieved by mechanically disrupting and reseeding organoids [69], microinjecting agents into the lumen [6], or exposing organoids before re-embedding them in Matrigel [26]. These procedures are technically demanding and may compromise structural integrity. Apical-out organoids are generated by removing the extracellular matrix and altering culture conditions to reverse polarity. Apical structures such as ZO-1 relocate to the external surface, allowing pathogens and experimental agents to act directly on the epithelial apex. PEDV and Senecavirus A (SVA) replicate more efficiently in apical-out organoids than in conventional basal-out cultures, supporting their use for receptor-dependent and apical-entry mechanisms [61,70].

Organoid-derived two-dimensional monolayers are also commonly used. Three-dimensional organoids are dissociated into single cells or small clusters and seeded on collagen IV- or Matrigel-coated Transwell membranes to form polarized epithelial layers. These monolayers retain organoid-derived epithelial characteristics while allowing direct measurement of TEER, permeability, and nutrient transport. They are therefore particularly suitable for studies of barrier injury and absorptive function [66,68,71]. The culture configurations are complementary and should be selected for the research question. Porcine intestinal organoids have been used to model diverse injuries, with pathogen infection receiving the greatest attention. Viral models include PEDV, TGEV, rotavirus, SVA, and human coronavirus OC43 and are used to examine tropism, replication, and host responses. Streptococcus suis, adherent-invasive Escherichia coli, and the intracellular pathogen Chlamydia suis have been used to study bacterial or intracellular pathogen interactions with the intestinal epithelium [26,43,61,68,69,70,71,72,73].

Organoids also model noninfectious injury. DON, T-2 toxin, other mycotoxins, and bacterial enterotoxins can be used to assess epithelial barrier and cellular dysfunction [6,43]. Hydrogen peroxide-induced oxidative injury and zinc-toxicity models address oxidative stress and metal exposure [65,74]. Combining organoids with gene editing further enables studies of genetic contributions to intestinal disease. Work in this area has so far focused mainly on colorectal cancer-related models, such as APC knockout and combined tumor-suppressor knockouts with KRAS activation [60,65,75]; although these cancer-oriented applications fall outside the scope of intestinal injury in piglets, the same gene-editing strategies, including use of the Rosa26 safe-harbor locus, can be applied to investigate host genetic determinants of susceptibility to intestinal injury.

Through different culture configurations and functional assays, organoid injury models partly overcome the limited cellular composition, spatial architecture, and tissue specificity of conventional two-dimensional cell lines. Each configuration has a distinct scope. Three-dimensional organoids are suited to tissue architecture and lineage studies, apical-out organoids to apical infection and receptor-dependent processes, and two-dimensional monolayers to barrier and drug assessments.

### 5.3. Treatment Methods for Organoid Injury Models

Viral infection protocols select an organoid configuration and inoculation strategy according to the route of viral entry. PEDV is commonly used in porcine intestinal organoid infection models. The PEDV DR13 strain can infect apical-out organoids [61]. For the PEDV-LJX strain, organoids are exposed at an MOI of 1 for 45 min and then re-embedded in Matrigel to preserve structure during infection [62]. The TGEV Miller strain can infect ileum-derived two-dimensional organoid monolayers at an MOI of 5 for 2 h to examine virus-epithelium interactions [68]. Porcine rotavirus requires protease activation and is commonly pretreated with 15 μg/mL trypsin at 37 °C for 1.5 h. Mixing virus with organoids before re-embedding yields higher infection efficiency [26]. Porcine organoids also support cross-species infection studies. Human coronavirus OC43 can complete its infection cycle after adsorption with 4 × 104 PFU at 33 °C for 2 h followed by re-embedding, producing infectious progeny. This finding indicates that porcine organoids may help investigate viral host adaptation [69]. SVA models use an MOI of 6 and a 2-h adsorption period to analyze replication and host responses [70].

Bacterial and intracellular pathogens are another important application. Streptococcus suis P1/7 can be applied to the apical surface of ileum-derived organoid monolayers. Co-culture at 1 × 10^6^ CFU/mL for 18 h allows assessment of transepithelial migration [43]. Adherent-invasive Escherichia coli induces extensive differential expression of inflammatory genes, including TNF and CXCL8, in colon- and ileum-derived monolayers, demonstrating the capacity of organoids to reproduce pathogen-induced host transcriptional responses [73]. ETEC enterotoxins can inhibit Wnt/β-catenin signaling and thereby impair intestinal stem-cell expansion [6]. Organoid infection has also served as an in vitro validation system for necroptosis observed in ETEC-challenged piglets [20]. Organoids are commonly used to model toxin- and physicochemical stress-induced epithelial injury. Mycotoxin studies often use subclinical concentrations close to field exposure. DON at 0.1 μmol/L and T-2 toxin at 0.01 μmol/L are applied alone or together for 24 h, after ATP-based viability assays identify a range without overt cytotoxicity [43]. For oxidative stress, treatment of piglet jejunal organoids with 1 mmol/L H_2_O_2_ for 3 days reduces budding and EdU-positive cells, increases ROS, and abnormally activates Wnt/β-catenin signaling [74]. Organoids also support evaluation of metal toxicity. After 30 min of zinc starvation, zinc acetate, ZnCl_2_, ZnSO_4_, or ZnO is applied at 10–200 μmol/L for 2–24 h to compare the effects of different zinc forms on intestinal epithelial function [65]. Porcine intestinal organoids support diverse pathogen, toxin, and inflammatory challenges and reproduce tissue-level epithelial injury more closely than conventional two-dimensional cell lines.

### 5.4. Assessment of Organoid Injury

Organoids more closely reproduce tissue architecture than conventional cell models, so injury assessment must integrate growth, barrier function, physiological activity, and molecular responses rather than relying on viability alone. Organoid-forming efficiency, budding rate, branch number, organoid number, and area distribution reflect stem-cell proliferation and structural maintenance. Machine-learning-assisted image analysis, including Cellpose with Fiji, enables automated segmentation and quantification of individual organoids and can improve accuracy and reproducibility over manual counting or a single endpoint viability assay [60]. EdU incorporation and Ki67 immunolabeling measure proliferative activity and epithelial regeneration after injury [26,74]. Barrier function is central, particularly in organoid-derived monolayers. Dynamic TEER measurements reveal differences in barrier development among intestinal regions. Ileal monolayers may reach peak TEER after approximately 36 h, whereas colonic monolayers can maintain approximately 187 Ω·cm^2^, and some collagen IV-coated systems reach approximately 700 Ω·cm^2^ [66,68,71]. Permeation of 4-kDa FITC-dextran measures paracellular leakage, and CFU counts after apical-to-basolateral bacterial migration quantify barrier defense during infection [43]. Organoids also permit assessment of epithelial physiology. Short-circuit current in Ussing chambers can measure SGLT1-mediated glucose absorption, CFTR-mediated ion secretion, and ENaC-mediated sodium absorption [71]. Fluorescent substrates such as 2-NBDG for glucose uptake and BODIPY-labeled fatty acids for lipid absorption assess nutrient uptake and metabolic sensing [33]. Lineage markers, pathogen replication, inflammation, and cell-death pathways provide additional outcomes. qPCR and immunofluorescence for LGR5, MUC2, CHGA, LYZ, and villin characterize stem-cell maintenance and epithelial lineages. In viral models, genome RNA copy number, TCID_50_, and viral-protein immunofluorescence quantify replication, whereas IFN-α, TNF-α, and IL-6 indicate host responses [26]. ROS assays, FDA/PI staining, and PI/Hoechst staining evaluate oxidative injury and cell-death patterns [60,69,74]. Omics approaches increasingly resolve organoid injury mechanisms. Bulk RNA sequencing with differential-expression, Gene Ontology, and KEGG enrichment analyses identifies region-specific responses and effects of host genetics [61,64,73]. Single-cell lineage tracing can monitor cell populations dynamically. Organoids from LGR5-H2B-GFP reporter pigs enable direct observation of intestinal stem-cell maintenance and differentiation [75]. Specialized functional tests address particular biological questions. Withdrawal of Wnt, R-spondin, and other growth factors can reveal dependence on stem-cell-maintenance signals and changes in transformation or stemness [60]. Organoid assessment has therefore progressed from single endpoints to integrated morphological, functional, molecular, and omics measures.

### 5.5. Current Technical Bottlenecks of Porcine Organoids

Although organoids reproduce aspects of intestinal tissue structure and function, important limitations remain. They consist mainly of epithelial cells and lack immune cells, vascular endothelial cells, nerves, smooth muscle, and commensal microorganisms. They therefore cannot fully reproduce multicellular immune regulation or complex disease mechanisms. These limitations directly affect the interpretation of organoid studies: infection models cannot reproduce neutrophil recruitment or systemic immune amplification, microbiota-dependent injury cannot be modeled without deliberate microbial co-culture, immune-mediated injury requires added immune components, and nutrient-absorption or post-weaning adaptation studies must account for the fetal-to-neonatal maturity of organoid epithelium. Research questions that depend on these components therefore require higher-complexity co-culture systems or in vivo validation. Recent approaches are beginning to close this gap. Co-culture of organoids with microorganisms, supported by apical-out polarity reversal and organoid-derived monolayers that expose the apical surface, already allows direct analysis of host-microbe interactions [26,43,61,70,72], and both co-culture with immune cells and microfluidic gut-on-a-chip systems, which are emerging in other species, are beginning to be adapted to porcine organoids. Their developmental trajectory resembles late fetal to neonatal intestine more closely than adult tissue. Region-specific expression also weakens to varying degrees during in vitro culture, and developmental phenotypes associated with the period before and after weaning remain difficult to reproduce consistently.

Standardization and long-term maintenance also limit broad application. Some wild-type porcine colonic and duodenal organoids expand for only three to five passages. Reported long-term cultures last approximately three to six months, and continuous provision of Wnt-related factors is considered a major constraint on sustained expansion. Commercial media, conditioned media, and recombinant growth-factor systems coexist, while donor age, culture conditions, and passage strategies differ among studies. These differences impair model stability and interlaboratory comparability.

**Table 3:** Comparison of models in porcine intestinal organoids.

Injury Type	Culture System	Dose	Time	Main Outcomes	Reference
PEDV infection	Duodenum/jejunum/ileum of 1-week-old piglets, apical-out 3D	PEDV strain DR13	1 h adsorption, sampled at 24 h	Viral loads were significantly higher in jejunal and ileal organoids than in duodenal organoids, with the strongest antiviral and IFN response in the jejunum	[61]
PEDV infection	Small intestine of 2-month-old specific-pathogen-free piglets	PEDV strain LJX, MOI = 1	45 min adsorption	PEDV N protein confirmed by Western blot and strainer-based immunofluorescence	[62]
Rotavirus infection	Jejunum of 1-day-old neonatal piglets, 3D (IntestiCult)	porcine rotavirus activated with 15 μg/mL trypsin	1.5 h activation, 3 h infection	Efficient replication in both 3D and 2D cultures was confirmed by growth kinetics; IL-6 and IFN-α increased significantly	[26]
TGEV infection	Ileum of Wuzhishan miniature pigs, 2D monolayer	TGEV strain Miller, MOI = 5	2 h infection	Viral gRNA load rose continuously from 2 to 24 hpi, with the highest viral titer at 24 hpi	[68]
SVA infection	Jejunum of 7-day-old piglets, apical-out 3D	SVA strain LJ-2021, MOI = 6	2 h adsorption	Viral load increased significantly at 12 hpi and peaked in the supernatant at 48 hpi	[70]
Human coronavirus OC43 infection	Porcine intestinal crypts, 3D fragments mixed with inoculum	OC43, 4 × 10^4^ PFU	2 h adsorption at 33 °C, up to 3 d	Robust replication with infectious progeny	[69]
ETEC infection	Jejunal crypts of piglets, 3D	ETEC CVCC196	-	Organoid infection corroborated epithelial necroptosis	[20]
Intracellular bacterium (C. suis)	Jejunum of 3- to 6-week-old piglets, 2D monolayer	C. suis R19, 10^5^–10^9^ IFU/mL	800× *g* centrifugation for 60 min	Dose-dependent inclusion formation without completing the developmental cycle	[72]
Mycotoxins (DON/T-2)	Ileal organoid 2D monolayer	DON 0.1 μmol/L, T-2 0.01 μmol/L	24 h	Toxins alone did not affect viability or permeability; combined DON and T-2 exposure decreased TEER and increased S. suis translocation	[43]
Oxidative stress	Jejunum of piglets, 3D	H_2_O_2_ 1 mmol/L	3 d treatment starting 48 h after passaging	Budding rate, branching coefficient, and EdU incorporation decreased significantly; ROS levels increased; and aberrant Wnt/β-catenin activation disturbed proliferation and differentiation	[74]
Metal toxicity	Ileum with Peyer’s patches of 26-week-old slaughter pigs, 3D + 2D	Zinc acetate/ZnCl_2_/ZnSO_4_/ZnO 10–200 μmol/L	2–24 h	At 24 h, only 200 μmol/L zinc acetate/ZnCl2/ZnSO4 severely disrupted monolayers, whereas ZnO had no effect	[65]

## 6. Conclusions and Perspectives

Research on intestinal injury in piglets now uses a complementary hierarchy of porcine intestinal epithelial cell lines, porcine intestinal organoids, and in vivo piglet models. Cell lines are inexpensive, high throughput, and readily manipulated, making them useful for initial mechanistic screening and evaluation of functional interventions. Their main limitations are simple cellular composition and unstable physiological phenotypes. Organoids retain regional identity, donor genetic background, and several epithelial lineages and therefore support tissue-specific and cell-interaction studies. They still lack an immune microenvironment, remain incompletely mature, and have limited culture standardization. In vivo piglet models provide the greatest physiological and production relevance but are costly, lengthy, multifactorial, and difficult to use for causal attribution. For practical application, we recommend a stepwise sequence in which the research question first defines the model level: cell lines such as IPEC-J2 are used for high-throughput mechanistic screening and for defining dose-time relationships; organoids or organoid-derived monolayers are then used when epithelial diversity, donor background, or tissue architecture is relevant; and in vivo piglet models finally validate physiological effects and production relevance. Model selection should weigh experimental control, physiological relevance, cellular complexity, immune and microbial interactions, throughput, cost, mechanistic manipulability, and the specific research question for which each model is most appropriate. It is also important to distinguish model establishment from model validation. Successful induction of cytokine elevation, ROS production, TEER reduction, or histological damage demonstrates model induction and phenotype confirmation, but does not by itself prove that the model reproduces a biologically relevant intestinal injury; validation additionally requires reproducibility across experiments and, where possible, correspondence with an in vivo or clinically relevant phenotype. Standardization is the main priority for improving comparability among studies. In vivo studies should report piglet age, sex and genetic background, intestinal segment, challenge strain or toxin and its purity, dose, administration route, exposure duration, sampling time, and injury endpoints. Cell studies should report passage number, seeding density, medium and serum, membrane type, and TEER measurement conditions. Organoid studies should report donor age and tissue source, passage number, medium and matrix composition, and culture configuration. Future work should improve model standardization and strengthen translation from in vitro findings to in vivo effects. Advances in organoid-immune cell-microorganism co-culture and spatial omics should support a multilevel validation research framework.

## Figures and Tables

**Table 2:** Comparison of porcine intestinal epithelial cell injury models.

Injury Type	Model	Dose	Time	Injury Outcomes	Reference
LPS	IPEC-J2	LPS 10 μg/mL	2–6 h	TNF-α and IL-8 secretion and NF-κB p65 phosphorylation significantly increased; TEER not significantly reduced within 6 h	[36]
LPS	IPEC-J2	LPS 10 μg/mL	48 h	Apoptosis significantly increased; caspase-3/8/9, IL-1β and TNF-α upregulated; partially reversed by porcine β-defensin 129	[37]
LPS	IPEC-J2	LPS 10 μg/mL	16 h	Transcriptome: MAPK/NF-κB pathway genes dominated the LPS response; dose-dependently reversed by sea buckthorn polysaccharide	[38]
LPS	IPEC-J2	LPS 1 μg/L	6 h	GPX4/SLC7A11 downregulated with ferroptosis induced; suppressed by I3C via the AhR-Nrf2 axis	[39]
LPS	IPEC-J2	LPS 40 μg/mL	48 h	Cell viability reduced to approximately 80%	[11]
LPS	IPEC-J2	LPS O149:B4 10 μg/mL	6 h	Pro-inflammatory factors IL-1β, IL-6 and TNF-α overexpressed; inhibited by oleanolic acid via TGR5	[15]
Mycotoxin (DON)	IPEC-J2	DON 0.5–2 μmol/L	24 h	Tight junction proteins were downregulated, paracellular permeability increased, and PPARγ transcriptional activity was suppressed	[13]
Mycotoxin (DON)	IPEC-J2	DON 0.5 μg/mL	24 h	Cell viability reduced below 70%; oxidative injury alleviated by selenomethionine via Nrf2	[40]
Mycotoxin (ZEA)	IPEC-J2	ZEA 20 μg/mL	24 h	ROS-positive cells increased more than tenfold; mitochondrial apoptosis and NF-κB/NLRP3 inflammation activated	[41]
Mycotoxin (FB_1_)	IPEC-J2	FB_1_ 20 μmol/L	48 h	Dose-dependent decrease in cell viability and TEER; increased apoptosis; tight junction proteins downregulated	[42]
ETEC infection	IPEC-J2	ETEC F4 10^6^ CFU/mL	3 h	TEER decreased and TLR4-NF-κB activated; dose-dependent protection by Bacillus licheniformis PF9	[44]
ETEC infection	IPEC-J2	ETEC 10^5^–10^8^ CFU/well	1–3 h	High doses (≥10^6^ CFU/well) significantly reduced cell viability; IL-1β and TNF-α upregulated	[45]
ETEC infection	IPEC-J2	ETEC (ATCC 25922)	1 h	IL-8, IL-6, IL-1β and TLR4/5/7 expression upregulated; p-p65 increased; reversed by porcine β-defensin 2	[46]
ETEC infection	IPEC-J2	ETEC-K88 MOI 10–100	12–24 h	Occludin significantly downregulated (MOI 10); bacterial adhesion rose after 4 h and peaked at 24 h	[48]
ETEC infection	IPEC-1	ETEC O9:H9 ~1 × 10^7^ CFU/mL	2 h	Infection successfully established; encapsulated phage reduced the bacterial load on cells	[34]
Bacterial infection (CPB2)	IPEC-J2	CPB2 toxin 20 μg/mL	24 h	Inflammatory injury with upregulated cytokines	[49]
Bacterial infection (CPB2)	IPEC-J2	CPB2 toxin 20 μg/mL	24 h	Epithelial apoptosis increased and caspase-3 upregulated, regulated by FTO-mediated m^6^A demethylation	[50]
Viral infection (TGEV/PEDV)	IPEC-J2	TGEV/PEDV MOI 0.1	24–48 h	Brush-border membrane NHE3 distribution reduced; NHE3 activity and Na^+^ uptake decreased	[25]
Viral infection (PEDV)	IPEC-J2	PEDV MOI 1	1–24 h	mtDNA release activated NLRP3; caspase-1 activity and IL-1β secretion increased; mtROS activated NF-κB	[59]
Oxidative stress (H_2_O_2_)	IPEC-J2	H_2_O_2_ 1 mmol/L	1 h	Cell viability reduced to 53%; soybean peptides protective via the Nrf2 pathway	[51]
Oxidative stress (H_2_O_2_)	IPEC-J2	H_2_O_2_ 0.4 mmol/L	1 h	Cell viability reduced to 52%; mitochondrial and death-receptor apoptotic pathways activated in parallel	[52]
Oxidative stress (tert-butyl hydroperoxide)	IPEC-J2	tert-butyl hydroperoxide 200 μmol/L	3 h	Cell viability decreased significantly, and ROS content increased	[53]
Oxidative stress (Fatty acids)	IPEC-J2	Fatty acids 200 μmol/L (n-6∶n-3 1∶1–20∶1)	-	Lipid peroxidation and ferroptosis aggravated with increasing n-6∶n-3 ratio	[55]
Environmental stress (Heat)	IPEC-J2	41 °C culture	5 d	Barrier function and mucus secretion impaired during heat stress	[57]
Nutritional stress (hyperosmolarity)	IPEC-J2	NaCl +150 mmol/L (600 mOsm)	2–24 h	ROS increased; mitochondrial apoptosis and autophagy enhanced; reversed by betaine	[58]
Nutritional stress (dietary antigen)	IPEC-J2	Soybean 7S globulin 5 mg/mL	24 h	Cell viability highly significantly reduced; NF-κB/MAPK pathway markers upregulated	[56]
Chemical injury (DSS)	IPEC-J2	DSS 0.08%	24 h	Cell viability dropped to 73% of control	[27]

## Data Availability

The original contributions presented in this study are included in the article. Further inquiries can be directed to the corresponding author.

## Abbreviations

The following abbreviations are used in this manuscript:

AhR	Aryl hydrocarbon receptor
ATP	Adenosine triphosphate
CAT	Catalase
CFU	Colony-forming unit
CHGA	Chromogranin A
CPB2	CLOSTRIDIUM perfringens beta2 toxin
CXCL8	C-X-C motif chemokine ligand 8
DON	Deoxynivalenol
DQ	Diquat
DSS	Dextran sulfate sodium
DTT	Dithiothreitol
DUOX2	Dual oxidase 2
EDTA	Ethylenediaminetetraacetic acid
ESC	Embryonic stem cell
ETEC	Enterotoxigenic Escherichia coli
FB_1_	Fumonisin B_1_
FITC	Fluorescein isothiocyanate
FTO	Fat mass and obesity-associated protein
GPX4	Glutathione peroxidase 4
GSH-Px	Glutathione peroxidase
H_2_O_2_	Hydrogen peroxide
IAP	Intestinal alkaline phosphatase
IFN	Interferon
IFU	Inclusion-forming unit
IL	Interleukin
IPEC-1	Intestinal porcine epithelial cell line 1
IPEC-J2	Intestinal porcine epithelial cell line J2
i-FABP	Intestinal fatty acid-binding protein
iPSC	Induced pluripotent stem cell
Ki67	Marker of proliferation Ki-67
LBW	Low birth weight
LGR5	Leucine-rich repeat-containing G-protein-coupled receptor 5
LPS	Lipopolysaccharide
LYZ	Lysozyme
MAPK	Mitogen-activated protein kinase
MDA	Malondialdehyde
MOI	Multiplicity of infection
MUC	Mucin
MyD88	Myeloid differentiation primary response 88
NF-κB	Nuclear factor kappa-light-chain-enhancer of activated B cells
NHE3	Sodium/hydrogen exchanger 3
NLRP3	NOD-like receptor family pyrin domain-containing 3
Nrf2	Nuclear factor erythroid 2-related factor 2
PAS	Periodic acid-Schiff
PCNA	Proliferating cell nuclear antigen
PEDV	Porcine epidemic diarrhea virus
PFU	Plaque-forming unit
PPARγ	Peroxisome proliferator-activated receptor gamma
ROS	Reactive oxygen species
S100A9	S100 calcium-binding protein A9
SGLT1	Sodium-glucose cotransporter 1
SLC7A11	Solute carrier family 7 member 11
SOD	Superoxide dismutase
SVA	Senecavirus A
TCID_50_	50% tissue culture infectious dose
TEER	Transepithelial electrical resistance
TGEV	Transmissible gastroenteritis virus
TGF-β	Transforming growth factor beta
TLR	Toll-like receptor
TNF-α	Tumor necrosis factor alpha
ZEA	Zearalenone
ZO-1	Zonula occludens-1
